# Persistent Off‐Season Dysregulation of Memory B Cell Subsets in Allergic Rhinitis

**DOI:** 10.1002/clt2.70100

**Published:** 2025-09-12

**Authors:** Maryam Jafari, Eric Hjalmarsson, Laila Hellkvist, Eirini Paziou, Agnetha Karlsson, Susanna Kumlien Georén, Lars‐Olaf Cardell

**Affiliations:** ^1^ Division of ENT Diseases Department of Clinical Science Intervention and Technology Karolinska Institutet Stockholm Sweden; ^2^ Department of Otorhinolaryngology Karolinska University Hospital Stockholm Sweden

**Keywords:** allergic rhinitis, biomarkers, immune dysregulation, memory B cells, minimal persistent inflammation

## Abstract

**Introduction:**

Allergic rhinitis (AR) is a common allergic airway disease. Although B cells play essential roles in AR pathogenesis, their subset distribution outside the allergen exposure period remains poorly characterized.

**Objective:**

To profile peripheral blood B cell subsets in AR patients during the pollen‐free season and compare them with healthy controls (HC), aiming to identify persistent immunological alterations and potential biomarkers.

**Methods:**

Peripheral blood mononuclear cells (PBMCs) were collected from a total of 28 participants, 14 patients with allergic rhinitis (AR) and 14 healthy controls (HC) during the off‐season. B cell subsets were identified using flow cytometry based on IgD and CD27 expression, classifying cells as naïve (IgD^+^CD27^−^), unswitched memory (IgD^+^CD27^+^), switched/conventional memory (IgD^−^CD27^+^), and unconventional memory B cells (IgD^−^CD27^−^). CD38 and CD24 were utilized to further distinguish transitional, naïve, memory, and plasma cell phenotypes. Immunoglobulin isotypes (IgG1‐4, IgA1^+^/IgA2^+^) were assessed specifically within conventional memory B cells, while CD86 expression was evaluated on IgM^+^ memory‐like and naïve B cells. Additionally, kappa (*κ*) and lambda (*λ*) light chain usage was analyzed to assess light chain distribution.

**Results:**

AR patients displayed lower frequencies of IgG1^+^, IgG2^+^, and IgA1^+^/IgA2^+^ memory B cells, along with elevated frequencies of IgG4^+^ and κ^+^ B cells. Additionally, CD86^+^IgM^+^ memory‐like B cells were significantly reduced in AR, suggesting altered activation dynamics. No significant differences were observed in CD24/CD38 profiling.

**Conclusion:**

Even outside allergen exposure, AR patients exhibit systemic B cell dysregulation, characterized by skewed class switching, altered subset distribution, and reduced activation markers expression. These findings underscore persistent immune imbalance in AR, identify potential off‐season biomarkers of allergic inflammation.

## 1 Introduction

Allergic rhinitis (AR) is a prevalent chronic inflammatory disease of the upper airways, characterized by IgE‐mediated responses to environmental allergens [1]. While the role of T‐helper type 2 (Th2) cells and IgE in the pathogenesis of AR is well established, increasing evidence suggests that B cells play a broader and more complex role in allergic disease [2, 3]. Beyond IgE production, B cells contribute to immune memory, class‐switch recombination, cytokine secretion, and antigen presentation, all of which can shape the progression of allergic inflammation [3]. Despite their critical involvement, the composition and functional dynamics of B cell subsets in AR, particularly during pollen‐free periods, remain incompletely understood (Table 1).

**TABLE 1:** Patient characteristics.

Characteristic	Patients (*N* = 14)	Healthy controls (*N* = 14)
Age^a^	38 (18–57)	31 (19–54)
Gender, *n* (%)		
Male	5 (36%)	6 (43%)
Female	9 (64%)	8 (57%)
Allergy		
Only birch, *n* (%)	0	N/A
Only timothy, *n* (%)	2 (14%)	N/A
Both birch and timothy, *n* (%)	12 (86%)	N/A

^a^ Median (range), N/A, not applicable.

Importantly, off‐season profiling of B cells provides an opportunity to examine baseline immune alterations that persist outside periods of active allergen exposure, potentially reflecting long‐term immune imprinting, disease chronicity, or biomarkers relevant for prediction and prevention.

B cell development begins in the bone marrow, where hematopoietic progenitor cells differentiate into naïve B cells through immunoglobulin gene rearrangement [4, 5]. These naïve B cells express membrane‐bound immunoglobulins, primarily IgM and IgD, and exit into the bloodstream [6]. Upon encountering antigen in secondary lymphoid organs, such as lymph nodes, B cells can become activated with the help of T follicular helper (Tfh) cells [7]. Activated B cells differentiate along two major pathways: some become memory B cells capable of rapid reactivation, while others develop into plasma cells that secrete large quantities of antibodies [4, 8]. These plasma cells may home to peripheral tissues or return to the bone marrow, contributing to long‐term humoral immunity [9].

The characterization of B cell subsets relies on the analysis of immunoglobulin isotypes and surface markers [6, 10]. IgG subclasses (IgG1, IgG2, IgG3, and IgG4) perform distinct immunological functions: IgG1 and IgG3 are potent activators of complement and Fc receptor pathways, IgG2 primarily targets polysaccharide antigens, and IgG4 is associated with chronic antigen exposure and tolerance, particularly in allergic diseases [11, 12, 13]. Similarly, IgA exists in two isoforms, IgA1 and IgA2, both essential for mucosal immunity through neutralization of allergens at epithelial surfaces [14, 15]. The kappa (*κ*) and lambda (*λ*) light chains offer insight into B cell repertoire diversity, with deviations in the κ:λ ratio suggesting clonal expansions or chronic immune stimulation [16]. Markers such as IgD and CD27 distinguish naïve (IgD^+^CD27^−^) from memory (CD27^+^) B cells, identifying antigen experience [17, 18]. CD86 expression marks B cell activation, while CD24, another important surface molecule, is involved in the regulation of inflammatory responses through immune checkpoint‐like functions [19]. Profiling these immunoglobulin subclasses and phenotypic markers provides a comprehensive framework to understand B cell dysregulation in allergic diseases such as AR [20].

Addressing the gaps in our understanding of B cell development and function in AR, particularly during pollen‐free periods, is crucial for identifying persistent immune imbalances that may serve as biomarkers or therapeutic targets in allergic disease management.

## 2 Materials and Methods

### 2.1 Patient Material

Peripheral blood was collected from a total of 28 individuals, including clinically diagnosed AR patients (*n* = 14) and healthy controls (HC; *n* = 14), outside the pollen season. All participants were sampled at least 2 months prior to the start of the birch and grass pollen season, ensuring a pollen‐free baseline for immune profiling. Participants met the inclusion criteria of having a confirmed history of seasonal AR due to birch and/or timothy pollen, a positive skin prick test, and an allergen‐specific IgE level of at least 0.35 kE/L in blood. Exclusion criteria comprised recent upper respiratory infections (within the past 2 weeks), recent use of local or systemic corticosteroids (within the past 2 months), antihistamine intake within 24 h prior to enrollment.

### 2.2 Ethical Statement

All participants provided written informed consent. The study was approved by the Swedish Ethical Review Authority (Diary Nos. 2021‐00325 and 2021‐06514‐02) and conducted in accordance with the Declaration of Helsinki. All procedures, including the handling of patient data, were performed in compliance with these ethical guidelines.

### 2.3 Flow Cytometry Panel

Peripheral blood mononuclear cells (PBMCs) were isolated by standard density gradient centrifugation. B cells were subsequently isolated from PBMCs using negative selection with the B Cell Isolation Kit II (Miltenyi Biotec, #130‐091‐151) and LS magnetic columns. Briefly, 10 million PBMCs were incubated with a biotin‐conjugated antibody cocktail and anti‐biotin microbeads to label non‐B cells. The labeled cells were retained on the LS column in a magnetic field, while unlabeled CD19^+^ and CD20^+^ B cells were collected in the flow‐through. To maximize B cell yield, columns were washed three times. The magnetically labeled non‐B cells were eluted separately and stored.

Isolated B cells were resuspended in DMEM and transferred to tubes for staining. To minimize non‐specific binding, cells were pre‐incubated with human Fc receptor blocking reagent (BD Biosciences, Cat# 564765) according to the manufacturer's instructions. Cells were then surface stained using a flow cytometry panel specified in Supporting Information S1: Table 1, which included markers for B cell identity (CD19, CD20), maturation (CD24, CD38, CD27, CD86, CD138), immunoglobulin isotypes (IgM, IgD, IgE, IgA1/IgA2, IgG1‐4), and light chains (*κ*, *λ*). After staining, cells were washed and fixed in 1% paraformaldehyde. Live/dead cell discrimination was performed using BD Horizon Fixable Viability Stain 780 (BD Biosciences).

Samples were acquired using the LSR Fortessa X20 flow cytometer (BD Biosciences) with standardized setting. On average, approximately 730,000 total events were recorded per sample, with 10,000–30,000 CD19^+^ B cells included in the final analysis. Data were analyzed using FlowJo v10 (BD Biosciences).

### 2.4 Statistical Analysis

Data were analyzed using GraphPad Prism software v10 (San Diego, California). All comparisons were performed between two independent groups (AR vs. HC). Prior to group comparisons, data were tested for normality and log‐normality using built‐in normality tests in GraphPad Prism. For normally distributed or log‐normal data, unpaired *t*‐tests were used; otherwise, the non‐parametric Mann‐Whitney *U* test was applied. Data are shown as individual values with mean ± SD. For visualization, 0 values in log‐scaled graphs were replaced with 0.0001. Statistical significance was set at *p* < 0.05.

## 3 Results

**FIGURE 1:**
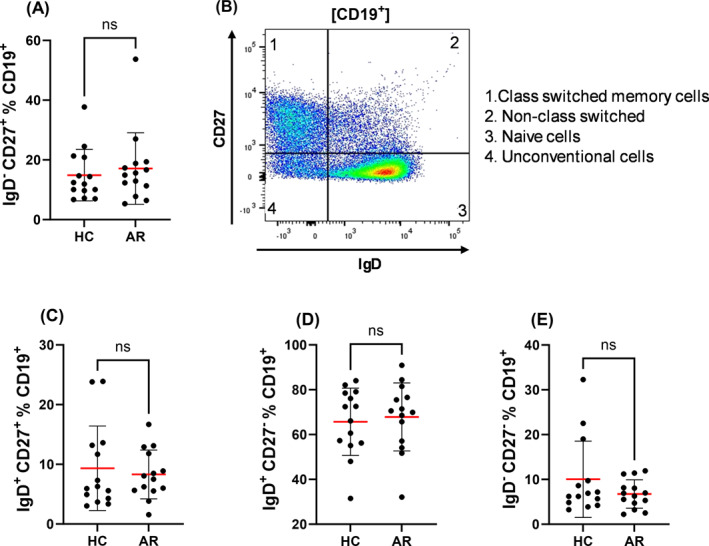
Distribution of peripheral B cell subsets in healthy controls (HC) and allergic rhinitis (AR) patients outside the pollen season. (A) The gating strategy identifying: Q1. Class‐switched memory B cells (IgD^−^CD27^+^), Q2. Unswitched (non‐class‐switched) memory B cells (IgD^+^CD27^+^), Q3. Naïve B cells (IgD^+^CD27^−^), Q4. Unconventional memory B cells or double‐negative B cells (IgD^−^CD27^−^). Panels show frequencies of each subset: (B) Class‐switched memory B cells (IgD^−^CD27^+^), (C) Unswitched memory B cells (IgD^+^CD27^+^), (D) Naïve B cells (IgD^+^CD27^−^), (E) Unconventional/double‐negative B cells (IgD^−^CD27^−^) Frequencies are expressed as a percentage of total CD19^+^ B cells. Each dot represents one individual donor (AR: *n* = 14; HC: *n* = 14). Red lines indicate group means; error bars represent mean ± standard deviation (SD). No statistically significant differences were observed between groups (unpaired *t*‐test; ns = not significant).

We first evaluated the distribution of peripheral B cell subsets, including conventional memory (IgD^−^CD27^+^) (Figure 1A; gating strategy shown in Figure 1B), unswitched memory (IgD^+^CD27^+^) (Figure 1C), naïve (IgD^+^CD27^−^) (Figure 1D), and unconventional memory populations (IgD^−^CD27^−^) (Figure 1E). No significant differences were observed between AR patients and healthy controls (HC) during the pollen‐free period.

**FIGURE 2:**
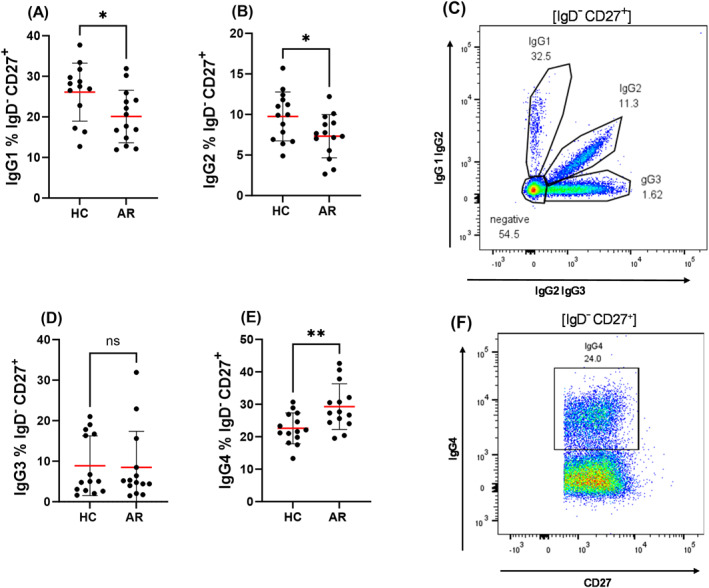
IgG subclass distribution in conventional memory B cells (IgD^−^CD27^+^) from healthy controls (HC) and allergic rhinitis (AR) patients. (A, B, D & E) Flow cytometric analysis of IgG subclass expression among conventional memory B cells (IgD^−^CD27^+^) in peripheral blood. Frequencies are shown for: (A) IgG1^+^, (B) IgG2^+^, (D) IgG3^+^, (E) IgG4^+^ B cells (all expressed as % of IgD^−^CD27^+^ B cells). Panels (C) and (F) the gating strategies used to identify IgG1‐3 (C) and IgG4 (F) expression. Gating was applied within the IgD^−^CD27^+^ population. Each dot represents one individual (HC: *n* = 14; AR: *n* = 14). Red lines indicate mean values; error bars show mean ± SD. *p < 0.05, **p < 0.01, ns = not significant (unpaired *t*‐test).

**FIGURE 3:**
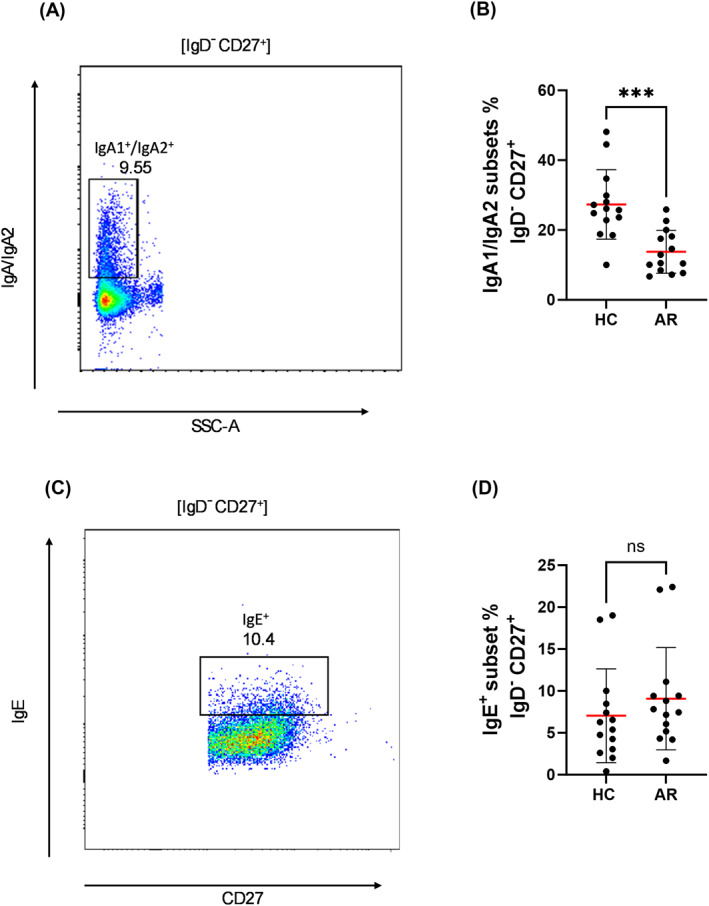
Frequencies of IgA1^+^/IgA2^+^ and IgE^+^ memory B cells in allergic rhinitis (AR) patients and healthy controls (HC). (A) The gating strategy for identifying IgA1^+^/IgA2^+^ cells within IgD^−^CD27^+^ memory B cells. (B) IgA1^+^/IgA2^+^ B cells within the IgD^−^CD27^+^ memory B cell compartment are significantly reduced in AR patients compared to HC. (C) The gating strategy for identifying IgE^+^ cells within IgD^−^CD27^+^ memory B cells. (D) Frequencies of IgE^+^ memory B cells (IgD^−^CD27^+^) do not differ significantly between groups. Each dot represents an individual donor (HC: *n* = 14; AR: *n* = 14). Bars represent mean ± SD. ***p < 0.001; ns = not significant (unpaired *t*‐test).

Next, we analyzed immunoglobulin subclass expression within conventional memory B cells. AR patients exhibited significantly lower frequencies of IgG1^+^ (Figure 2A) and IgG2^+^ (Figure 2B) cells, accompanied by a marked increase in IgG4^+^ cells (Figure 2E). IgG3 expression remained unchanged (Figure 2D). Additionally, IgA1^+^ and IgA2^+^ memory B cells were reduced in AR patients (Figure 3A), while IgE^+^ memory B cell frequencies did not differ between groups (Figure 3D).

**FIGURE 4:**
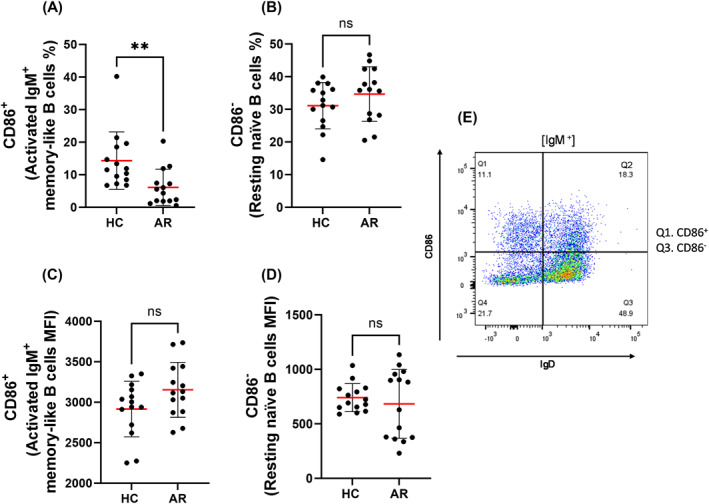
Differential expression of activation marker CD86 on IgM^+^ memory‐like and naïve B cells in allergic rhinitis (AR) patients and healthy controls (HC). (A) The frequency of CD86^+^ cells within the IgM^+^ memory‐like B cell population was significantly lower in AR patients compared to HC. (B) No significant difference was observed in the frequency of CD86^−^ naïve B cells between groups. (C) Mean fluorescence intensity (MFI) of CD86 on IgM^+^ memory‐like B cells showed a trend toward higher expression in AR patients. (D) CD86 MFI on naïve B cells was similar between groups. (E) The gating strategy for CD86 versus IgD expression on B cells. Each dot represents one donor (HC: *n* = 14; AR: *n* = 14). Bars indicate mean ± SD. **p < 0.01; ns = not significant (unpaired *t*‐test).

CD86 expression was assessed to evaluate B cell activation. The frequency of CD86^+^ IgM^+^ memory‐like B cells was significantly reduced in AR patients compared to HC (Figure 4A), while no significant difference was observed in the frequency of CD86^−^ naïve B cells (Figure 4B). However, mean fluorescence intensity (MFI) analysis showed a trend toward increased CD86 expression on IgM^+^ memory‐like B cells in AR patients (Figure 4C), despite their lower frequency, whereas CD86 MFI on naïve B cells remained unchanged between groups (Figure 4D).

**FIGURE 5:**
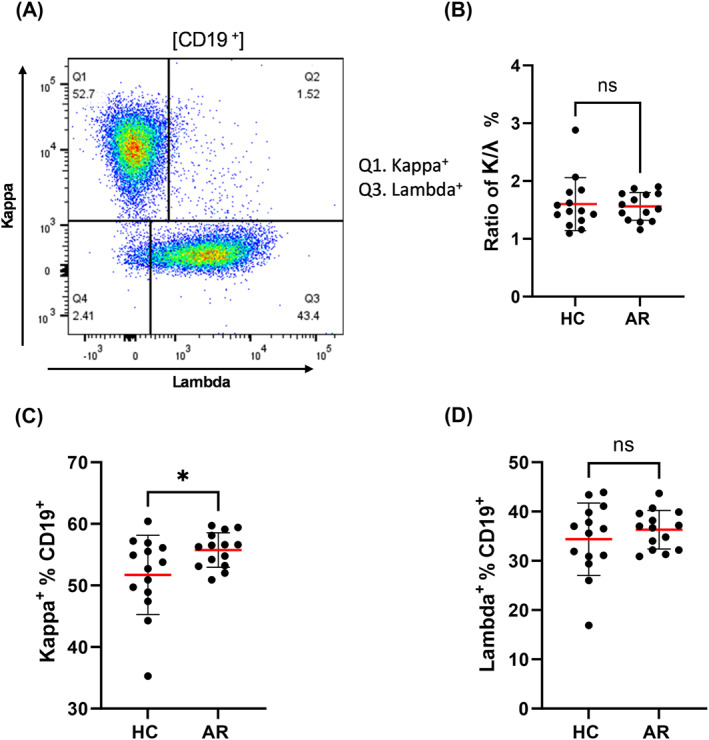
Kappa (*κ*) and lambda (*λ*) light chain expression in peripheral B cells from healthy controls (HC) and allergic rhinitis (AR) patients. (A) The gating strategy for *κ* versus *λ* light chain expression on B cells. Region 1 represents κ^+^ B cells, and region 3 represents λ^+^ B cells. (B) The *κ*/*λ* ratio shows no significant difference between groups. (C) The frequency of κ^+^ B cells is significantly higher in AR compared to HC. (D) The frequency of λ^+^ B cells show no significant difference between groups. Each dot represents an individual donor (HC: *n* = 14; AR: *n* = 14). Bars represent mean ± SD. *p < 0.05; ns = not significant (unpaired *t*‐test).

Additionally, the analysis of the κ^+^/λ^+^ ratio revealed no significant differences between HC and AR (Figure 5B). In contrast, light chain analysis indicated a higher frequency of κ^+^ B cells in AR patients (Figure 5C), whereas no significant differences were observed for λ^+^ B cells (Figure 5D). To clarify this we note that the increased frequency of κ^+^ B cells did not significantly alter the *κ*:*λ* ratio, likely due to a concurrent but non‐significant increase in λ^+^ B cells.

Detailed gating strategies can be found in Supporting Information S1: Figures 1–7.

## 4 Discussion

In this study, we profiled peripheral B cell subsets and immunoglobulin subclasses in patients with AR and HC during the off‐season. The overall distribution of major B cell populations including naïve, unswitched memory, switched memory, and unconventional double‐negative B cells was comparable between AR patients and HC. However, specific dysregulations within the memory B cell compartment were observed.

In this study, we focused our isotype analysis on conventional class‐switched memory B cells (IgD^−^CD27^+^) due to their established role in recall responses and immunoglobulin class‐switch recombination. This subset provides the most biologically relevant context for studying antigen‐experienced cells producing IgG subclasses, IgA, and IgE. Analyzing isotypes in unconventional memory B cells (IgD^−^CD27^−^) was avoided to minimize background and uncertainty, given their low frequency, undefined function, and limited marker resolution in our dataset. However, we acknowledge that this subset may still contribute to allergic immune dysregulation and should be considered in future investigations.

AR patients exhibited reduced frequencies of IgG1^+^ and IgG2^+^ conventional memory B cells, alongside the frequency of IgG4^+^ memory B cells were increased. Memory B cells expressing IgA1^+^/IgA2^+^ were also decreased.

Additionally, a subset of IgM^+^ memory‐like B cells expressing the activation marker CD86 was significantly lower in AR patients, while the frequency of naïve CD86^−^ B cells remained unchanged. A higher prevalence of *κ* light chain positive B cells was observed, with no significant difference in λ^+^ B cells. Finally, CD38/CD24 profiling revealed no significant differences in B cell subset distribution.

Although the proportions of major B cell subsets between AR and HC appeared preserved, this likely reflects the resting state of allergen‐driven immune processes during the off‐season [8]. However, previous studies, such as those by Luo et al., reported an increase in CD27^+^ memory B cells and plasma cells, accompanied by a decrease in naïve B cells outside the pollen season, suggesting that subtle immune alterations may persist [19]. Variations across studies may stem from differences in cohort size or phenotyping strategies. Despite the apparent stability in naïve/memory B cell distribution, functionally relevant subsets may still be dysregulated [21]. Thus, AR patients may retain normal quantities of memory B cells overall, but these cells may exhibit a phenotype skewed toward a pro‐allergic state [8, 21].

We further identified skewing within the IgG subclass composition of memory B cells. Frequencies of IgG1^+^ and IgG2^+^ memory B cells were reduced, while IgG4^+^ memory B cells were increased (no difference was noted in IgG3^+^ memory cells). IgG1 and IgG2 are typically dominant in responses to protein and polysaccharide antigens, and their reduction may reflect immune deviation or exhaustion of classical memory responses [11]. In contrast, IgG4 is associated with chronic antigen exposure and tolerance [22]. Previous studies have demonstrated that successful allergen immunotherapy (AIT) increases IgG4^+^ memory B cells and serum IgG4 levels, correlating with clinical improvement, as shown by Heeringa et al. [23]. Although no participants in this study were undergoing active AIT, we cannot fully exclude the possibility of prior or unreported exposure to immunotherapy, which may have influenced the observed IgG4^+^ memory profile. In untreated AR patients, the elevated IgG4^+^ memory subset likely reflects repeated seasonal allergen exposure driving a partial immunoregulatory adaptation [24]. As highlighted by Qin et al., IgG4 can act as a “double‐edged sword” in allergic diseases, providing protective blocking effects while simultaneously marking chronic allergen stimulation [25]. Our findings align with this interpretation, suggesting that IgG4^+^ memory development in AR may serve as a compensatory but incomplete countermeasure to ongoing allergic inflammation.

A reduction in IgA1^+^/IgA2^+^ memory B cells was also observed in AR patients. IgA plays a critical role in immune exclusion at mucosal surfaces, and its deficiency has been associated with increased allergic susceptibility [26, 27]. Our findings are consistent with those reported by Zheng et al., where circulating IgA^+^ memory B cells were reduced in AR patients and restored following AIT [28]. IgA memory cells correlated positively with IL‐10^+^ regulatory T cells and inversely with symptom severity, supporting a regulatory role in allergic inflammation [29]. The impaired generation or maintenance of mucosal IgA memory in AR may reflect a failure in TGF‐β or IL‐10 signaling, favoring a Th2‐driven IgE and IgG4 response instead [30]. These results highlight a potential immunological gap in AR, with important implications for disease susceptibility and persistence. This IgA deficiency may also compromise mucosal barrier integrity or alter microbial homeostasis, potentially contributing to persistent immune dysregulation in AR. The concept of minimal persistent inflammation describing ongoing mucosal inflammation even outside allergen exposure may help explain our findings. The observed B cell alterations, despite the absence of pollen, suggest a lasting immunological imprint that could contribute to persistent nasal sensitivity and subclinical inflammation, as described in perennially symptomatic allergic individuals [31].

B cell activation status, assessed via the costimulatory molecule CD86, revealed a reduction in CD86^+^ IgM memory‐like B cells in AR patients, while CD86^−^ naïve B cells were unaffected. CD86 is critical for antigen presentation and T cell co‐stimulation [32]. Although the decrease in CD86^+^ memory B cells may appear paradoxical in an inflammatory disease, it likely reflects either the off‐season resting state of allergen‐specific B cells or a regulatory adaptation [32]. Previous studies have shown that allergen exposure selectively induces CD86 expression in B cells from atopic individuals, suggesting that AR B cells are primed for activation upon stimulation but remain inactive outside the pollen season [32]. Alternatively, repeated allergen exposure could induce an exhausted or anergic memory B cell phenotype. Moreover, Yao et al. reported that memory B cells in AR display aberrant CD23 expression, promoting IgE‐mediated antigen presentation over classical CD86 CD28 co‐stimulation [33]. Collectively, these findings suggest that allergen‐specific B cells in AR may favor alternate activation routes and altered interactions with T cells.

An elevation in κ^+^ light chain‐expressing B cells have been observed in AR patients, while no significant difference in the *κ*: *λ* light chain ratio was found between the groups. Under normal conditions, this ratio approximates 2:1, reflecting random light chain rearrangement [34]. Recent studies have demonstrated that free immunoglobulin light chains themselves can function as inflammatory mediators. Specifically, *κ* light chain proteins have been shown to activate mast cells and contribute to allergic inflammation independently of IgE [34]. Elevated serum levels of free *κ* light chains, correlating with eosinophilic inflammation, have been reported in patients with severe asthma by Basile et al. Similarly, Powe et al. observed increased free light chains in nasal secretions of individuals with allergic rhinitis and identified Igκ/Igλ‐positive cells in nasal mucosa, primarily consisting of mast cells and plasma cells [35]. These findings support the hypothesis that the *κ*‐biased B cell response observed in AR may contribute to local inflammation through mast cell activation, independent of IgE [16]. This mechanism represents a potentially novel pathway for immune activation in AR and warrants further investigation.

Finally, CD38 and CD24 expression profiling were performed using a previously established gating strategy and revealed no significant differences in B cell subset distribution between AR patients and HC [36]. The frequencies of memory B cells (CD38^−^CD24^+^), which are important for maintaining long‐term immunological memory, were comparable between groups, suggesting that the memory B cell compartment is not markedly altered in AR. Similarly, transitional B cells (CD38^+^CD24^+^), representing immature B cells recently emigrated from the bone marrow, as well as naïve B cells (CD38^+^CD24^−^), showed no significant differences, indicating that early B cell development and peripheral entry remain largely preserved in AR. Plasma cells (CD38^++^CD24^−^), which reflect terminally differentiated antibody‐secreting cells, also did not differ significantly between groups [8, 37]. These findings suggest that, based on CD24/CD38 profiling, the major stages of B cell maturation and differentiation are maintained in AR patients, with no clear shift toward activation or terminal differentiation detectable in peripheral blood outside the allergen exposure season.

## 5 Study Limitation

While our findings provide valuable insights into memory B cell dysregulation in off‐season AR, several limitations must be acknowledged. First, the modest sample size may limit statistical power and generalizability. Subtle differences in B cell subsets could have gone undetected, and larger cohorts are needed to validate these observations. Second, the cross‐sectional design limited the assessment of dynamic changes between pollen and off‐season periods; longitudinal studies would offer deeper insights. Third, although our phenotypic characterization was comprehensive, flow cytometry does not assess B cell functions such as cytokine production or antigen specificity, which limits mechanistic interpretation.

We also acknowledge that IgE^+^ memory B cells are extremely rare and technically challenging to detect by flow cytometry. Our use of direct surface staining without enrichment or in vitro stimulation may have led to an overestimation of their abundance due to low event counts and potential background staining. Finally, serum immunoglobulin subclass levels and cytokine profiles were not assessed in parallel, which could have provided additional systemic context.

## 6 Conclusion

In summary, patients with allergic rhinitis exhibit persistent alterations in their memory B cell compartment even during the off‐season. These include a shift toward IgG4 memory, reduced IgG1^+^, IgG2^+^ and IgA^+^ memory B cells, decreased activation marker expression on IgM memory‐like cells, and a *κ*:*λ* light chain imbalance. Together, these features suggest lasting immune imprinting that may shape future allergen responses. Clinically, B cell profiling could support biomarker development for disease monitoring and therapeutic targeting. Future allergen immunotherapy strategies might benefit from approaches that enhance IgA memory, restore IgG subclass balance, and promote regulatory B cell function. These findings emphasize the relevance of B cell‐oriented approaches in the management of allergic rhinitis.

## Author Contributions

**Maryam Jafari:** conceptualization, investigation, writing – original draft, methodology, validation, visualization, writing – review and editing, software, formal analysis, project administration, data curation, resources. **Eric Hjalmarsson:** investigation, visualization, writing – review and editing, supervision, methodology, project administration, resources. **Laila Hellkvist:** resources, writing – review and editing. **Eirini Paziou:** resources, writing – review and editing. **Agnetha Karlsson:** resources, writing – review and editing. **Susanna Kumlien Georen:** conceptualization, investigation, methodology, project administration, writing – review and editing, validation, supervision, visualization. **Lars‐Olaf Cardell:** conceptualization, investigation, funding acquisition, methodology, validation, visualization, project administration, supervision, writing – review and editing.

## Conflicts of Interest

The authors declare no conflicts of interest.

## Supporting information


Supporting Information S1

## Data Availability

Researchers interested in the data generated and analyzed in this study may request access from the corresponding author.
