# Supplementary material for: Persistent Off‐Season Dysregulation of Memory B Cell Subsets in Allergic Rhinitis
Source: Clin Transl Allergy. 2025 Sep 12;15(9):e70100. doi: 10.1002/clt2.70100 (PMC12426901; doi:10.1002/clt2.70100)
Supplement: Supplementary file 1 — Supporting Information S1 [file CLT2-15-e70100-s001.docx]

**Supplementary Table 1.** Flow cytometry antibody panel used for the identification and characterization of total and allergen-specific B cells.

| **Laser** | **Fluorochrome** | **Marker** | **Clone** | **Volume Used (µL, Dilution)** | **Manufacturer** |
| --- | --- | --- | --- | --- | --- |
| 355 nm (UV) | BUV395 | IgM | G20-127 | 4 µL (1:25) | BD Biosciences |
|  | BUV496 | IgE | G7-26 | 5 µL (1:20) | BD Biosciences |
|  | BUV805 | CD20 | 2H7 | 5 µL (1:20) | BD Biosciences |
|  | BV421 | Biotin (Allergen) | REA746 | 2 µL (1:50) | Milteny Biotec |
| 405 nm (Violet) | BV510 (V500) | CD19 | HIB19 | 2 µL (1:50) | BD Biosciences |
|  | BV650 | CD24 | ML5 | 4 µL (1:25) | BD Biosciences |
|  | BV711 | CD38 | HIT12 | 3 µL (1:33) | BD Biosciences |
|  | BV786 | CD3 | SP34-2 | 2 µL (1:50) | BD Biosciences |
| 488 nm (Blue) | FITC / BB515 | IgG2, IgG3 | SAG2/SAG3 | 2 µL (1:50) | Cytognos |
|  | PE | IgG1, IgG2 | SAG1/SAG2 | 2 µL (1:50) | Cytognos |
|  | PE-CF594 | CD138 | MI15 | 3 µL (1:33) | BD Biosciences |
|  | PerCP-Cy5.5 | IgA1, IgA2 | SAA1/SAA2 | 2 µL (1:50) | Cytognos |
| 561/594 nm | PE-Cy7 | CD27 | M-T271 | 4 µL (1:25) | BD Biosciences |
| 633/640 nm (Red) | APC | IgA1, IgA2 | SAA1/SAG4 | 2 µL (1:50) | Cytognos |
|  | APC-H7 | IgD | IA6-2 | 4 µL (1:25) | BD Biosciences |
|  | BV605 | Kappa light chain | G20-193 | 1 µL (1:100) | BD Biosciences |
|  | AF-700 (R718) | Lambda light chain | 1-155-2 | 1 µL (1:100) | BD Biosciences |

The supplementary table 1 lists laser lines, fluorochromes, markers, antibody clones, volumes used per 100 µL staining volume, calculated dilutions, and manufacturers. The panel also included reagents for the detection of allergen-specific B cells using biotinylated tetramers; however, these data are not presented in the current manuscript due to low event counts outside the pollen season. Clone names and antibody dilutions were included to improve reproducibility.

**Supplementary Figures 1-7**

Supplementary Figures 1 through 7 illustrate the flow cytometry gating strategies used to identify and characterize peripheral blood B cell subsets and their immunoglobulin expression profiles, corresponding to main manuscript Figures 1 through 5. CD19⁺ B cells were consistently gated from PBMCs by sequential selection of lymphocytes, singlets, and viable cells. B cell subsets were defined based on IgD and CD27 expression into class-switched memory, unswitched memory (IgM⁺), naïve, and double-negative memory B cells. The subsequent panels demonstrate the expression of IgG subclasses (IgG1, IgG2, IgG3, IgG4; Supplementary Figs. 2–3), IgA1/IgA2 (Fig. 4), IgE (Fig. 5), IgM⁺ memory B cells (Fig. 6), and κ/λ light chains (Fig. 7) within relevant B cell compartments.


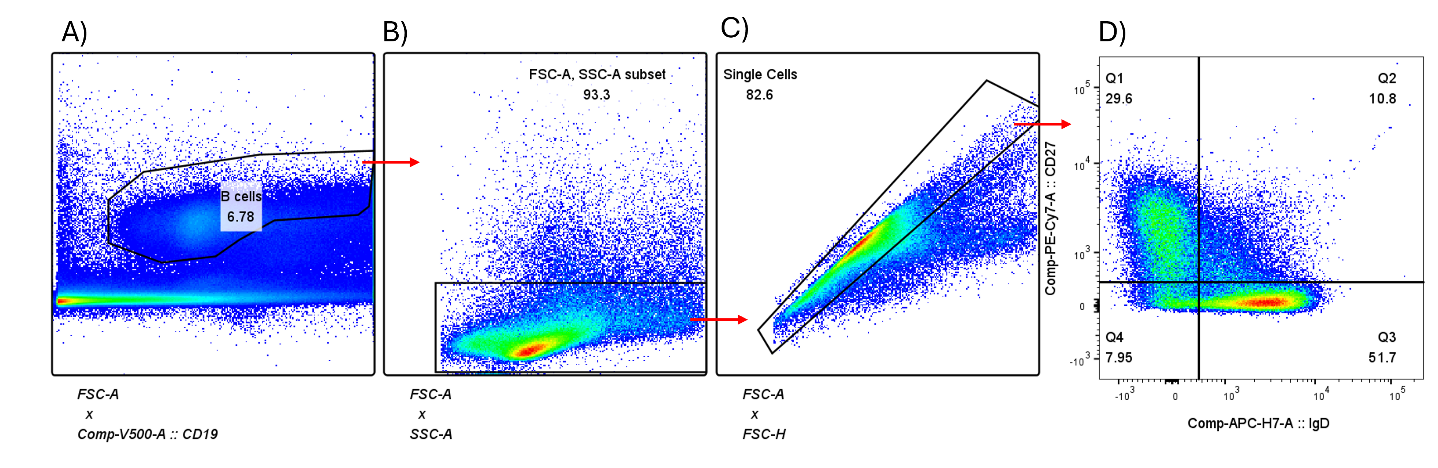
**Supplementary Figure 1:** Gating strategy for the identification and characterization of peripheral blood B cell subsets by flow cytometry. CD19⁺ B cells were first identified by gating on lymphocytes in FSC-A vs CD19 (panel A), followed by gating on the PBMC population using FSC-A vs SSC-A (panel B). Singlets were selected based on FSC-A vs FSC-H (panel C). In panel D, B cell memory subsets were classified based on IgD and CD27 expression: IgD⁻CD27⁺ (class-switched memory B cells), IgD⁺CD27⁺ (unswitched/IgM memory B cells), IgD⁺CD27⁻ (naïve B cells), and IgD⁻CD27⁻ (unconventional/double-negative memory B cells).


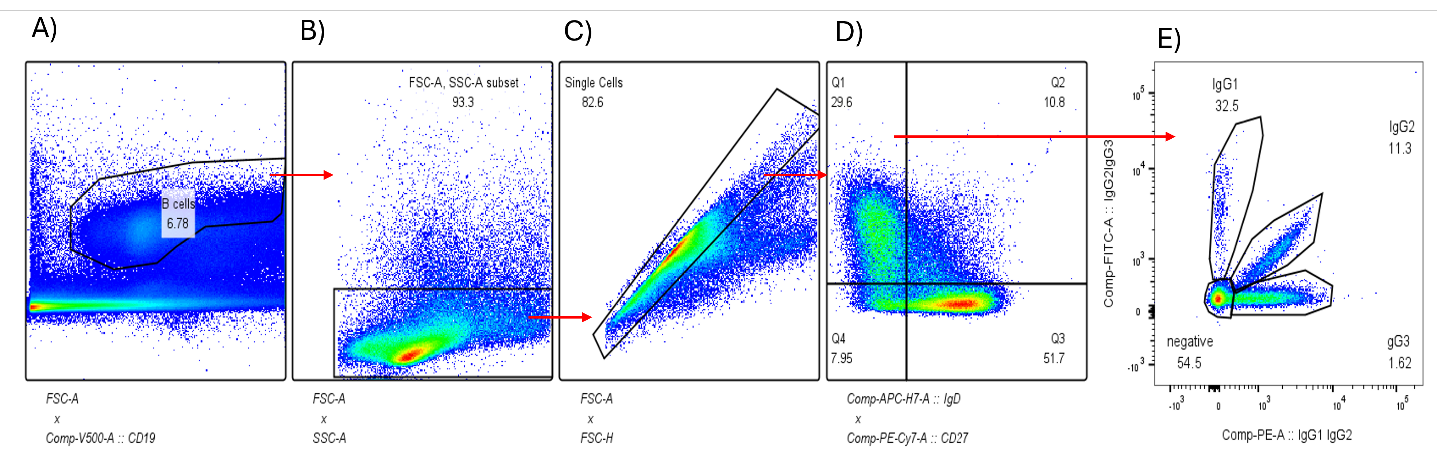


**Supplementary Figure 2.** Gating strategy for the identification and characterization of peripheral blood B cell subsets by flow cytometry. CD19⁺ B cells were first identified by gating on lymphocytes in FSC-A vs CD19 (panel A), followed by gating on the PBMC population using FSC-A vs SSC-A (panel B). Singlets were selected based on FSC-A vs FSC-H (panel C). In panel D, B cell memory subsets were classified based on IgD and CD27 expression: IgD⁻CD27⁺ (class-switched memory B cells), IgD⁺CD27⁺ (unswitched/IgM memory B cells), IgD⁺CD27⁻ (naïve B cells), and IgD⁻CD27⁻ (unconventional/double-negative memory B cells).
Panel E shows the expression profiles of IgG1, IgG2, and IgG3 within the gated conventional memory B cell population (IgD⁻CD27⁺).


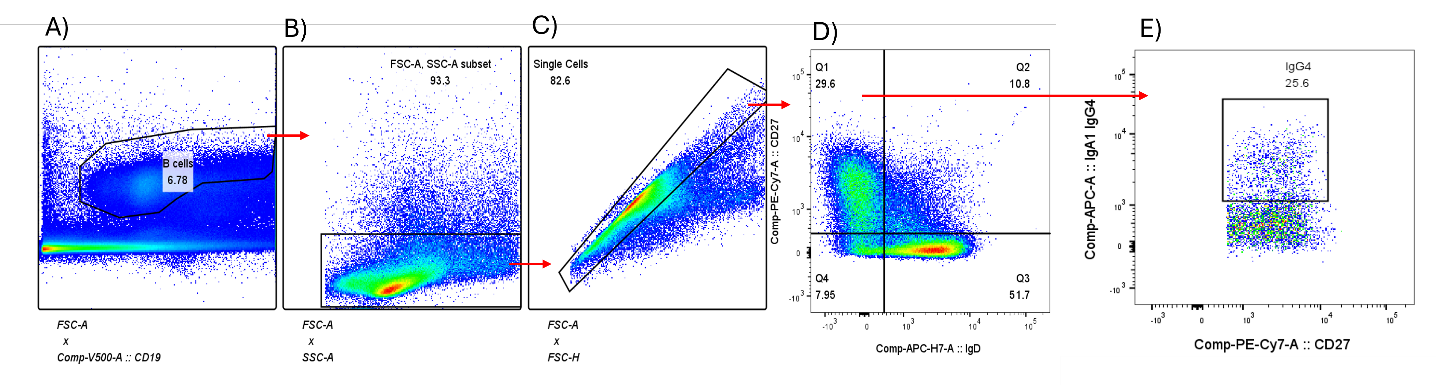


**Supplementary Figure 3.** Gating strategy for the identification and characterization of peripheral blood B cell subsets and IgG4 expression in conventional memory B cells. CD19⁺ B cells were gated from lymphocytes based on FSC-A vs CD19 (A), followed by selection of PBMCs using FSC-A vs SSC-A (B). Singlets were identified using FSC-A vs FSC-H (C). B cell memory subsets were defined based on IgD and CD27 expression (D), where IgD⁻CD27⁺ cells represent class-switched memory B cells, IgD⁺CD27⁺ are unswitched/IgM memory B cells, IgD⁺CD27⁻ are naïve B cells, and IgD⁻CD27⁻ represent unconventional or double-negative memory B cells. IgG4 expression was subsequently analyzed within the gated class-switched memory B cell population (E).


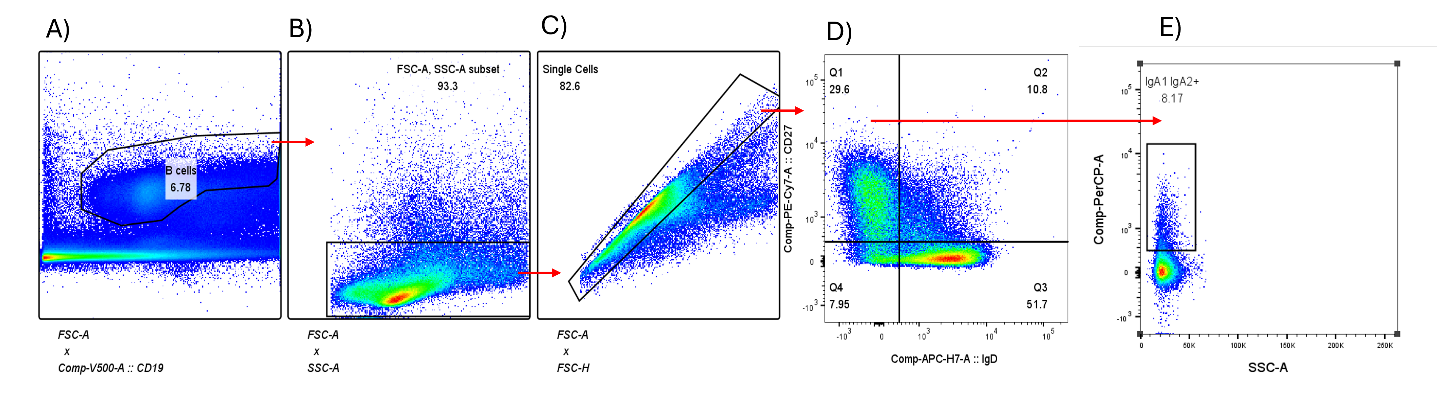


**Supplementary Figure 4.** Gating strategy for the identification and analysis of IgA1^+^/IgA2^+^-expressing class-switched memory B cells in peripheral blood. CD19⁺ B cells were first identified from lymphocytes based on FSC-A vs CD19 (A), followed by selection of the PBMC population using FSC-A vs SSC-A (B). Singlets were gated using FSC-A vs FSC-H (C). B cell subsets were classified based on IgD and CD27 expression (D), with class-switched memory B cells defined as IgD⁻CD27⁺. Within this gated population, surface expression of IgA1 and IgA2 was analyzed (E).


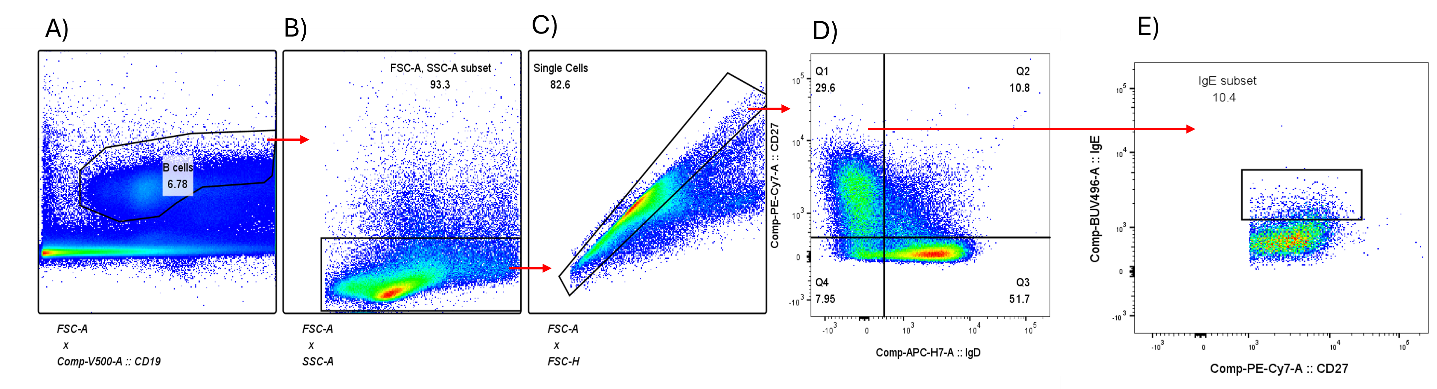


**Supplementary Figure 5.** Gating strategy for the identification of IgE⁺ memory B cells in peripheral blood. CD19⁺ B cells were first identified by gating on lymphocytes using FSC-A vs CD19 (A). PBMCs were selected based on FSC-A vs SSC-A (B). Singlets were gated using FSC-A vs FSC-H (C). Memory B cells were classified based on IgD and CD27 expression, with class-switched memory B cells defined as IgD⁻CD27⁺ (D). Within this population, IgE⁺ memory B cells were identified based on IgE and CD27 expression (E).


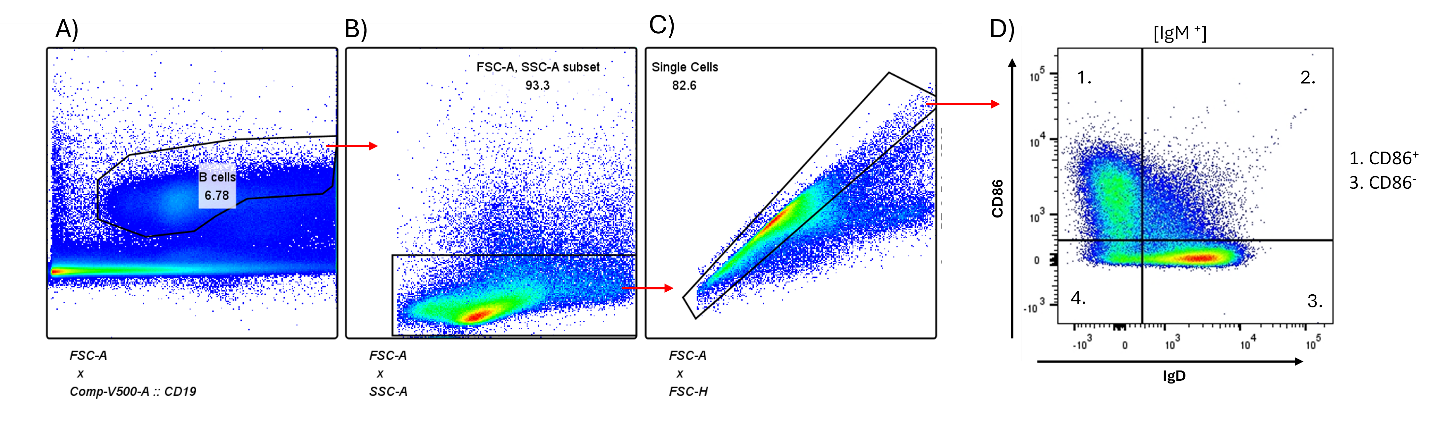


**Supplementary Figure 6.** Gating strategy for the identification of IgM⁺ memory B cells in peripheral blood. CD19⁺ B cells were first identified by gating on lymphocytes using FSC-A vs CD19 (A). PBMCs were selected using FSC-A vs SSC-A (B), and singlets were gated based on FSC-A vs FSC-H (C). Within the CD19⁺ population, IgM⁺ memory B cells were identified as IgD⁺CD27⁺ (D).


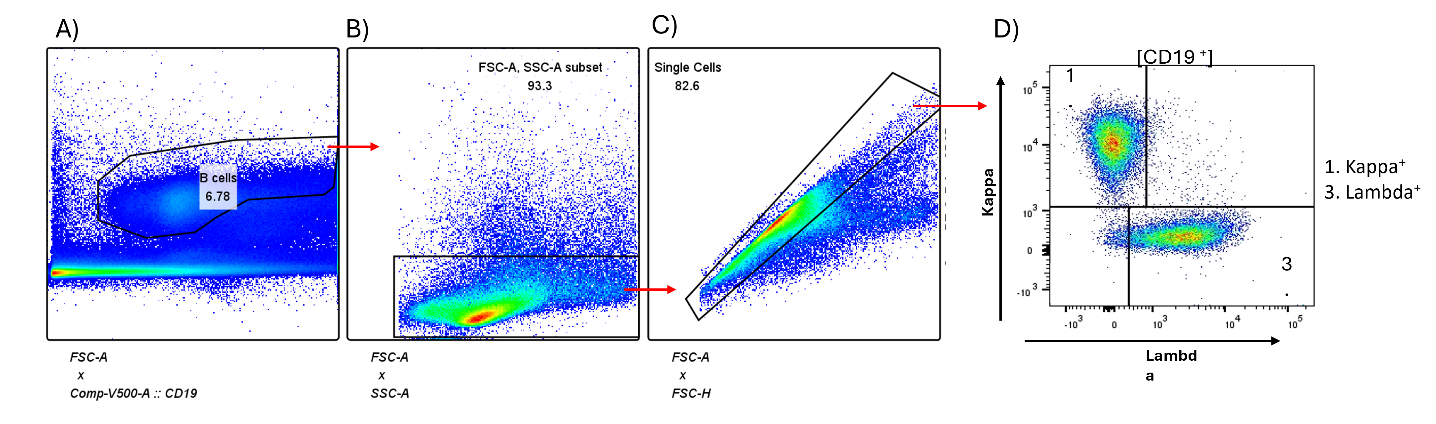


**Supplementary Figure 7.** Flow cytometry gating strategy for the analysis of immunoglobulin light chain expression in peripheral blood B cells. (A) CD19⁺ B cells were identified from lymphocytes using FSC-A vs CD19. (B) The PBMC population was gated based on FSC-A vs SSC-A. (C) Singlets were selected using FSC-A vs FSC-H. (D) Within the CD19⁺ B cell population, κ (kappa) and λ (lambda) light chain expression was assessed.
